# Chyloperitoneum Associated with Relapse of Nephrotic Syndrome

**DOI:** 10.1155/2012/961816

**Published:** 2012-12-03

**Authors:** Jui-Hsiang Lin, Wei-Jie Wang

**Affiliations:** ^1^Division of Nephrology, Department of Medicine, Taoyuan General Hospital, 1492 Chung-Shan Road, Taoyuan City, Taoyuan County 33004, Taiwan; ^2^Department of Biomedical Engineering, Chung Yuan Christian University, 200 Chung Pei Rood, Chung Li City, Taoyuan Country 32023, Taiwan

## Abstract

Chyloperitoneum associated with nephrotic syndrome is rare clinical entity and characterized by milky ascites. The modalities for detection of chyloperitoneum are laboratory investigations and radiological images. Lipoprotein electrophoresis is useful for confirmation of the diagnosis with chyloperitoneum. Treating underlying disease is the most important in the management of these patients.

## 1. Introduction 

Chyloperitoneum is a rare clinical entity. Nephrotic syndrome rarely causes chylous ascites. We report a rare case of chyloperitoneum in a young man with relapse of nephrotic syndrome. Detection of chylous ascites can be readily made with simple tests and radiological images. We present the figure of lipoprotein electrophoresis to disclose prominent elevation of chylomicrons. 

## 2. Case Report 

**Figure 1:**
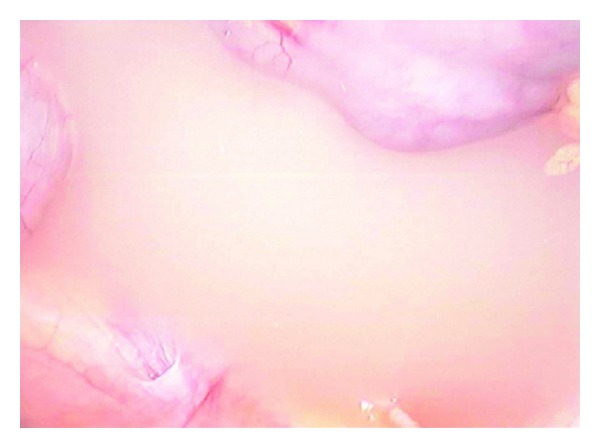
Diagnostic laparotomy revealed milky white ascites.

**Figure 2:**
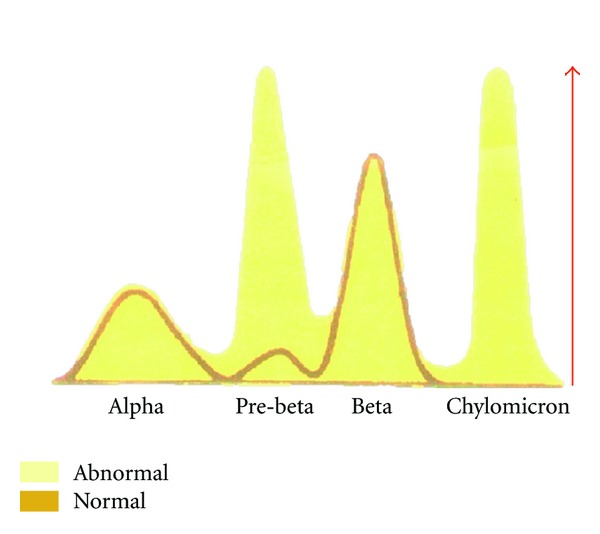
Lipoprotein electrophoresis showed predominant chylomicrons (arrow).

This 29-year-old man presented to our emergency department (ED) with one day of fever and sudden onset of diffuse abdominal pain which was poorly localized without any referred pain. He had medical condition including one year of minimal change nephrotic syndrome undergoing methylprednisolone 20 mg daily, and he never suffered any previous relapse of nephrotic syndrome. The patient's temperature was 38.3°C, blood pressure was 118/63 mmHg, heart rate was 118 beats per minutes, and he suffered weak pulsation, pitting edema at extremities, tenderness at the whole abdomen with diffuse abdominal guarding, and absence of bowel sounds. Laboratory studies revealed a hemoglobin concentration of 129 (normal 133–162) g/L, leukocyte count of 26.31 (normal 3.54–9.06) × 10^9^/L, platelet count of 260 (normal 165–415) × 10^9^/L, urinary protein excretion of 4.1 (normal less than 0.15) g/day, albumin of 15 (normal 40–50) g/L, total cholesterol of 324 (normal less than 200) mg/dL, and creatinine of 88 (normal 53–106) *μ*mol/L. An unenhanced computed tomography (CT) scan of the abdomen showed bilateral pleural effusion and massive ascites. Paracentesis was difficult to be done in the patient with peritoneal signs. The surgeon performed diagnostic laparotomy. The peritoneal fluid was milky white ascites (Figure 1) involving polymorphonuclear leukocyte count 5285 cell count/mm^3^ and triglyceride level of 208 mg/dL. He was diagnosed with spontaneous bacterial peritonitis and chyloperitoneum associated with nephrotic syndrome. On admission, he received intravenous ceftriaxone 2 g daily. Two days later, fever subsided, but chylous ascites did not improve. Ascetic fluid culture failed to grow. A lymphoscintigraphy of whole body revealed no definite evidence of lymph flow obstruction or leakage. Lipoprotein electrophoresis in chylous ascites showed the chylomicrons of 29.6% (normal 0–1.9%) (Figure 2). Prednisolone 60 mg daily was administrated. One week later, nephrotic syndrome improved, and chylous ascites dramatically disappeared. In three months of the followup visits, the clinical condition was relatively stable.

## 3. Discussion 

There are a lot of causes of chylous ascites. The most common causes are abdominal malignancy and cirrhosis. Other causes are infectious, congenital, inflammatory, postoperative, traumatic, and miscellaneous disorders including right heart failure, dilated cardiomyopathy, and nephrotic syndrome. The pathogenesis of chylous ascites in nephrotic syndrome remains unclear. Lindenbaum and Scheidt explained that hypoalbuminemia led to edema of the mucosa and submucosa in intestinal structures [1]. The change that increases the permeability of mucosal cells, submucosal lymphatics, or serosal lymphatics results in the leakage of chylomicrons into the peritoneal space [1]. 

Detection of chylous ascites can be readily made with simple tests and radiological images. The triglyceride levels in ascites are very important in defining chylous ascites and typically above 200 mg/dL [2]. Lipoprotein electrophoresis in chylous peritoneal fluid can be performed to show the presence of predominant chylomicrons. Computed tomography of abdomen is suggested to identify pathological intra-abdominal lymph nodes and masses. Other kinds of radiological studies such as lymphangiography and lymphoscintigraphy are recommended in evaluating abnormal retroperitoneal nodes, leakage from dilated lymphatics, fistulization, and obstruction of the thoracic duct. These radiological images may select patients who are candidates for surgical intervention [2]. 

In conclusion, chyloperitoneum associated with nephrotic syndrome is very rare disorder [3–5]. Lipoprotein electrophoresis is useful for confirmation of the diagnosis with chyloperitoneum. Minimal change nephrotic syndrome in the adults is characterized by a good response to corticosteroid. Treating underlying disease is the most importance in the management of these patients.
